# Influences of Maternal Factors Over Offspring Allergies and the Application for Food Allergy

**DOI:** 10.3389/fimmu.2019.01933

**Published:** 2019-08-23

**Authors:** Takashi Fujimura, Shelly Zing Chin Lum, Yuka Nagata, Seiji Kawamoto, Michiko K. Oyoshi

**Affiliations:** ^1^Division of Immunology, Boston Children's Hospital, Boston, MA, United States; ^2^Hiroshima Research Center for Healthy Aging (HiHA), Graduate School of Advanced Sciences of Matter, Hiroshima University, Higashi-Hiroshima, Japan; ^3^Division of Gastrointestinal Pathophysiology, Institute of Natural Medicine, University of Toyama, Toyama, Japan; ^4^Department of Pediatrics, Harvard Medical School, Boston, MA, United States

**Keywords:** allergen, asthma, breast milk, food allergy, immune complexes, immunoglobulins, *in utero*, environmental factors

## Abstract

The prevalence of food allergy has been steadily rising worldwide with the highest incidence noted among younger children, and increasingly recognized as a growing public concern. The first known ingestion of foods often causes allergic reaction, suggesting that sensitization of offspring with food allergens may occur during pregnancy and/or through breastfeeding. This creates a milieu that shapes the neonatal immune responses to these allergens. However, the effects of maternal allergen exposure and maternal sensitization with allergens on development of allergies in offspring remain controversial. This review discusses recent advances from human data in our understanding of how maternal factors, namely, food allergens, allergen-specific immunoglobulins, cytokines, genetics, and environmental factors transferred during pregnancy or breastfeeding influence offspring allergies and how such effects may be applicable to food allergy. Based on information obtained from mouse models of asthma and food allergy, the review also dissects the mechanisms by which maternal factors, including the impact of immune complexes, transforming growth factor-β, vitamin A, and regulatory T-cell responses, contribute to the induction of neonatal tolerance vs. development of allergic responses to maternally transferred allergens.

## Introduction

The rise in the prevalence of allergic diseases such as asthma, pollinosis, and food allergy has continued worldwide for more than 50 years and has become a global public health concern, especially in young children. Sensitization rate to one or several allergens among school children are approaching 40 to 50% worldwide (1). Consistently, a recent cohort study identified 32% of children in southern Sweden suffered with at least one allergic disease and 33% of them develop multiple allergies (2).

Food allergy currently affects 5–8% of the United States population (3). The prevalence of food allergy in young children could grow as rapidly as 1% in a decade (4). Diagnosis of food allergy by food challenge or a combination of history of clinical symptoms and indication of food-specific immunoglobulin (Ig) E by skin prick tests or blood tests (5) provide compelling data that the incidence of food allergy is increasing in both Western and developing countries (6). Such escalation in the prevalence of food allergy has been stated as the second wave of the allergy epidemic after asthma (6). Food allergy is adverse immune responses to normally harmless food allergens that result in clinical symptoms implicating the dermatologic, respiratory, gastrointestinal, cardiovascular, and neurologic systems. Eight major food allergens (milk, egg, peanut, tree nuts, wheat, soy, fish, and crustacean shellfish) (7) cause most of the serious food allergy reactions in the United States. Peanut is the most common food allergen in Europe and in the United States and its prevalence in young population under 18-year-old increased from 0.2% in 1997 to 1.1% in 2008 (8). Food allergy is the most common reasons for potentially life-threatening anaphylaxis in children. The numbers of visit to a large academic pediatric emergency department for food-induced anaphylaxis has been more than doubled from 2001 to 2006 (9). There are currently limited options of management and treatment for food allergy, including avoidance of causal allergic foods, treatment of accidental exposures with epinephrine, and allergen-specific immunotherapy (10), and additional therapeutic and preventive approaches are urgently needed.

The first known ingestion of foods often causes allergic reactions (11), suggesting that exposure of offspring to food allergens that shape neonatal immune system may occur during pregnancy and/or through breastfeeding. However, the effects of maternal factors on the development of food allergy in offspring are poorly understood. Epidemiological studies in humans have shown conflicting results regarding the role of maternal allergen exposure in the development of allergies in offspring. Past studies have identified an increased risk (12) or no association (13) of maternal peanut consumption with peanut sensitization in offspring. In contrast, maternal exposure and sensitization to food allergens could be protective of offspring from allergic diseases in humans and in mice (14–19). In this review, we highlight recent research advances in the understandings from mouse and human studies of protective and adverse impacts of maternal factors in the development of allergies and food allergy in children with a focus on maternal factors including allergens, immunoglobulins (Igs), immune complexes (IC), cytokines transferred from mothers to offspring *in utero* and via breast milk, together with genetic and environmental factors that could further facilitate the neonatal immune responses to allergens.

## Maternal Protective Influences Over Offspring Allergy

### Human Studies

Maternal allergen consumption during their pregnancy and breastfeeding has been thought to control allergen sensitization in offspring, because first contact to food allergens could occur *in utero* as major food allergens could appear in amniotic fluid in an intact form (20). Contrarily, maternal nutrition status, allergens, and Igs, transferred *in utero* and via breast milk may prevent allergic sensitization in children. Two decades ago, United Kingdom Government's Chief Medical Officer's Committee on Toxicity of Chemicals in Food, Consumer Products and the Environment (COT) recommended that atopic mothers should avoid consumption of peanut and peanut products during pregnancy and breastfeeding to prevent peanut allergy in offspring. Following this recommendation, however, the prevalence of peanut allergy in school-age children increased and even resulted in the highest prevalence of peanut allergy in 4- to 5-year-old children (21). These data indicate no significant preventive effect by maternal allergen avoidance. Further, maternal dietary restriction during pregnancy or breastfeeding that aimed to prevent offspring allergy did not show a significant protective effect, instead, resulted in a lower gestational weight gain or adverse effects in maternal nutrition and fetal growth (22, 23). More recent studies have implied that the effect of maternal diet should be considered together with postnatal introduction of food in offspring (24–26). These studies underscore the requirement of alternative strategies rather than maternal dietary antigen avoidance for the prevention of food allergy (Table 1). In this section, we focus on the effects of maternal nutrition *in utero* and via breastfeeding on prevention of allergies in children.

**Table 1:** Maternal and offspring food consumption and the outcomes in offspring allergy in human cohort studies.

**Readout**	**Population/size**	**Dose/duration/timing**	**Association/outcome**	**Effects**	**References**
***IN UTERO***					
Cow's milk allergy in children	6,288 children	During pregnancy	Association of high consumption of milk products during pregnancy with a lower risk of cow's milk allergy in offspring of non-allergic mothers	Protective	(27)
Peanut or Tree nuts allergy in offspring	8,205 children (10 to 14 years of age)	During pregnancy	· Association of maternal consumption of at least five servings of peanut/tree nuts per week with a lower prevalence of peanut or tree nut allergy in offspring of non-allergic mothers · No association of maternal consumption of peanut/tree nuts with the prevalence of peanut or tree nuts allergy in offspring of peanut/tree nuts-allergic mothers	· Protective (non-allergic mothers) · No association (allergic mothers)	(15)
Allergy and asthma in mid-childhood	1,277 mother-child pairs (7.93 ± 0.82 years of age)	During pregnancy	Association of higher maternal consumption of (1) peanut during the first trimester with a reduced risk of peanut allergic reaction, (2) milk during the first trimester with reduced asthma and allergic rhinitis, and (3) wheat during the second trimester with reduced atopic dermatitis in offspring	Protective	(14)
Peanut sensitization in offspring	503 infants (3 to 15 months of age) with likely milk or egg allergy without previous diagnosis of peanut allergy	· The first or second trimester of pregnancy or during breastfeeding · The third trimester of pregnancy · Never breastfed with frequent consumption of peanut during pregnancy	· No significant effect of maternal peanut consumption on offspring peanut sensitization · A dose-dependent association of maternal peanut consumption during the third trimester of pregnancy with peanut sensitization in offspring · A strong association of maternal peanut consumption with peanut sensitization in offspring	· No association · Allergic · Allergic	(12)
Peanut allergy	13,971 children of non-atopic and atopic mothers (1 to 18 months of age)	During pregnancy	No association of maternal peanut consumption during pregnancy with challenge-proven peanut allergy in preschool children	No association	(13)
Maternal avoidance and avoidance of solid and allergic food in childhood	288 infants of atopic parents (4, 12, and 24 months of age)	· Maternal food avoidance during the third trimester of pregnancy and breastfeeding period · Offspring avoidance of solid food for 6 months, avoidance of cow's milk, corn, citrus, and wheat for 12 months, and avoidance of egg, peanut, and fish for 24 months	· Association of maternal food avoidance with a lower cumulative prevalence of atopy in offspring at 12 months, occurring from reduced food-associated atopic dermatitis, urticaria, and/or gastrointestinal disease, as compared to offspring of mothers with unrestricted diets · Association of offspring food avoidance with a significant suppression of the prevalence of food allergy and milk sensitization before 2 years of age. · No association of offspring food avoidance with the prevalence in food allergy, atopic dermatitis, allergic rhinitis, asthma, any atopic disease, and serum IgE levels at 7 years of age	· Protective · Protective (before 2 years of age) · No association (at 7 years of age)	(28) (29)
**BREASTFEEDING**					
Peanut sensitization at 7 years of age	342 infants (7 years of age)	During breastfeeding and direct peanut introduction to infants	· Lowest prevalence of peanut sensitization in infants whose mothers consumed peanut while breastfeeding with an early direct introduction of peanut before 12 months of age · Increased risk of peanut sensitization in infants whose mothers consumed peanut while breastfeeding with delayed introduction of peanut beyond 12 months of age, or maternal peanut avoidance with an early direct introduction of peanut by 12 months of age	· Protective (infants breastfed with early introduction) · Allergic (infants breastfed with delayed introduction)	(26)
Milk sensitization	186 children (0 to 4 years of age)	Breastfeeding more than 6 months	Exclusive or partial breastfeeding for more than 6 months reduced milk sensitization in children at 1 and 1.5 years of age as compared to formula-fed children.	Protective	(30)
Food allergy	649 children with challenge-proven food allergy (5 to 214 months of age)	Direct breastfeeding or bottle-feeding	· Every additional month of breastfeeding decreased a risk for food allergy by ~4% per month. · No significant association between the prevalence of food allergy and direct breastfeeding vs. bottle-feeding	Protective	(31)
Childhood asthma	3,296 children (3 years of age)	· Direct breastfeeding · Indirect breastfeeding	· Direct breastfeeding was more protective against childhood asthma at 3 years of age as compared to formula-feeding. · Indirect breastfeeding (breastfeeding with some expressed breast milk) provided an intermediate protection as compared to direct breastfeeding.	· Protective · Protective	(32)
Offspring wheezing	2,773 infants (3, 6, 12 months of age)	Breastfeeding with maternal asthma	· Offspring wheezing was reduced by 62% with exclusive breastfeeding and by 37% with partial breastfeeding supplemented with complementary foods as compared to infants with no breastfeeding. · Offspring wheezing was not protected by partial breastfeeding supplemented with formula.	· Protective (exclusive and partial breastfeeding supplemented with foods) · No association (partial breastfeeding with formula)	(33)
**EARLY INTRODUCTION**					
Peanut allergy (LEAP)	834 infants (4 to 10 months of age) at high risk for allergy	Food introduction between 4 and 10 months of age	· Among the infants initially negative for peanut skin-prick test, the prevalence of peanut allergy at 60 months of age was lower in the consumption group than those in the avoidance group. · Among the infants initially positive for peanut skin-prick test, the prevalence of peanut allergy was lower in the consumption group than those in the avoidance group.	· Protective (skin-prick test negative infants) · Protective (skin-prick test positive infants)	(34, 35)
Food allergy (EAT)	1,303 infants (3 months of age)	Food introduction before 6 months of age	· Multiple allergenic foods can be safely introduced into infant diet without adverse influence on breastfeeding. · Significantly lower prevalence of any food allergy in the early introduction group in families that closely adhered to the challenging protocol	· Safe early introduction of allergenic foods · Protective	(36)

*EAT, Enquiring About Tolerance; LEAP, Learning Early About Peanut Allergy*.

#### *In utero* Factors

##### Food allergen consumption

Reducing the risk of allergy by dietary means is a logical response to the increase in food allergy and other allergic diseases. In contrast to maternal allergen avoidance, prenatal consumption of potentially allergenic foods has been shown to prevent allergic sensitization in children. A study enrolled 6,288 children in Finland showed an association between high ingestion of milk products during pregnancy and a lower risk of cow's milk allergy in children [odds ratio (OR), 0.56] (27). The preventive effects were observed in children of non-allergic mother (OR, 0.30). Maternal ingestion of milk products was correlated with levels of beta-casein-specific IgA in cord blood in children without cow's milk allergy. Consequently, the study suggested that maternal milk ingestion during pregnancy exhibits tolerogenic effects *in utero* especially in non-allergic mothers. In a recent prospective study with 8,205 children between 10- and 14-year-old, the prevalence of peanut or tree nuts allergy in offspring was lower in children of non-allergic mothers who ingested at least five servings of peanut/tree nuts per week during pregnancy (OR, 0.31) (15). However, there was no association of maternal consumption of peanut/tree nuts during pregnancy and the risk of peanut/tree nuts allergy in offspring of mothers who were allergic to peanut/tree nuts, indicating that this preventive effect may be operative in non-allergic mothers but not in allergic mothers (15). Another cohort study in United States enrolled 1,277 mother-child pairs reported that maternal diet during pregnancy was associated with decreased allergy and asthma in mid-childhood (mean age, 7.9-year-old) (14). Higher maternal consumption of peanut during the first trimester was associated with 47% reduced odds of peanut allergen reaction (OR, 0.53). Higher maternal milk ingestion during the first trimester was also associated with reduced risk of asthma (OR, 0.83) and allergic rhinitis (OR, 0.85). Maternal consumption of wheat during the second trimester was associated with reduced risk of atopic dermatitis (OR, 0.64). These results indicate that early encounter with food allergens through maternal diet during the critical period of fetal development during the first trimester may lead to tolerance rather than sensitization. Collectively, these studies suggest that maternal allergen consumption during pregnancy could be beneficial to prevent allergic sensitization in offspring.

##### Allergen-specific Igs

Placental transfer of maternal Igs is known to provide a protective passive immunity to the fetus against infection (37). The selective transport of maternal IgG to the fetus during pregnancy is mediated by the neonatal Fc receptor (FcRn) expressed in placental syncytiotrophoblast (38). Allergen-specific immunotherapy (AIT) appears safe in pregnant women (39) and placental transfer of allergen-specific IgG1 and IgG4 antibodies, but not IgE, from AIT-treated mothers to offspring has been shown (40). Consistently, children of AIT-treated mothers during pregnancy exhibited high levels of IgG antibodies and fewer positive skin prick test as compared with children of untreated mothers (41). Increased levels of cord blood IgG antibodies to cat and birch were associated with less atopic symptoms in the children of allergic mothers during the first 8 years of life (42). These results suggest that maternal allergen-specific IgG antibodies may reduce the development of offspring atopy, but further data are required regarding a potential protective role of placentally transferred allergen-specific IgG toward allergic diseases, particularly food allergy in offspring.

##### Microbiota

Over the last decade, the importance of commensal bacteria in the host immune responses has been increasingly recognized, particularly bacteria colonizing the gastrointestinal tract. The composition of intestinal microbiota in newborn infants is dependent on many factors, such as type of maternal deliveries. Cesarean delivery has been associated with the incidence of atopy and allergic diseases in children, including atopic dermatitis, rhinitis, asthma, and eosinophilic esophagitis (43–46). Through vaginal delivery, offspring ingest maternal vaginal and colonic microbiota with passage through the birth canal, which probably represents the most important phase of initial gut colonization. This exposure of newborn infants to maternal bacteria results in stimulation of toll-like receptor (TLR) and production of interleukin (IL)-12 and interferon (IFN)-γ, which promote the differentiation of naïve helper T (Th) cells into Th1 effector cells that secrete IFN-γ (44, 47). Cesarean section was associated with reduced pro-inflammatory cytokine responses to TLR1/2 stimulation, followed by an increased abundance of bacterial colonization in the airway during late infancy, therefore increasing the risk of infantile wheezing (48). Levels of transforming growth factor (TGF)-β1 were lower in newborns following cesarean section (49). Hence, newborns born through vaginal delivery and exposed to higher levels of TGF-β1 may exhibit more protection toward atopy at later life, potentially reflecting the capacities of TGF-β1 to skew the production of IgE toward non-inflammatory isotypes IgG4 and IgA (50). Additional long-term studies of investigating maternal microbiota and its relationship with infant microbiota, as well as the development of infant food allergy are needed.

##### Dietary supplements

Development of human immune system begins *in utero* and continues after birth (51). Nutrition influences the global health and disease of children in early life. During the last decade, many examples showed prevention of allergic sensitization in children by modifying maternal diet during pregnancy (52, 53), indicating an *in utero* programming of allergic susceptibility in offspring. Increased levels of 25-hydroxyvitamin D (25(OH)D) in cord blood have been inversely associated with the risk of asthma and wheeze during childhood (54). Rationally, the association of maternal vitamin D supplementation with the development of atopic diseases in offspring has been investigated. In Vitamin D Antenatal Asthma Reduction Trial (VDAART), 806 women between the gestational ages of 10 to 18 weeks were supplemented with daily vitamin D for 22 to 30 weeks (55). Maternal supplementation with high-dose (4,000 IU/d) vitamin D reduced the risk of asthma and recurrent wheezing in offspring by 3 years of age (56, 57). In Copenhagen Prospective Studies on Asthma in Childhood in 2010 (COPSAC2010), 623 pregnant women were administered daily 2,400 IU/d vitamin D3 from the gestational ages of 24 week to 1 week postpartum (58). Combined analysis of these two studies demonstrated 25% reduced risk of asthma and recurrent wheeze at 0–3 years of age (adjusted OR, 0.74; *p* = 0.02) by maternal vitamin D supplementation (59). Of note, high concentration of serum maternal 25(OH)D was associated with an increased incidence of allergic airway disease in offspring at 20–25 years of age (60). With regard to food allergy, vitamin D insufficiency early in life has been associated with the risk of food allergy (61). In addition, maternal prenatal vitamin D supplementation was inversely associated with the development of food allergy, especially in infants with polymorphisms that increases the biological availability of serum vitamin D (62). These heterogeneous findings may reflect the importance of context, timing, and dose of vitamin D in the actions on the offspring allergies and potential differences in the mechanisms of asthma and food allergy.

Early exposure of omega 6 (n-6) and omega 3 (n-3) polyunsaturated fatty acids (PUFAs), commonly found in many nuts, seeds, and vegetable oils, during pregnancy shows different capacities to alter the incidence of atopic sensitization and allergy in children (63). Arachidonic acid (20:4, n-6) is a catabolite of linoleic acid (18:2, n-6) and a main substrate for eicosanoids, which are a family of lipid mediators that induce inflammation (63, 64). Higher consumption of n-3, such as docosahexaenoic acid and eicosapentaenoic acid primarily found in certain fish, decreased the levels of inflammatory eicosanoids from arachidonic acid and increased the levels of alternative eicosanoids that have anti-inflammatory properties (63). Maternal intake of oily fish containing higher docosahexaenoic acid and eicosapentaenoic acid as well as fish oil supplements showed protective effects on wheeze and asthma in offspring (65). Similarly, maternal fish oil supplementation reduced the severity of atopic eczema and the risk of egg sensitization in 1-year-old offspring (66). High maternal intake of α-linoleic acid (18:3), another n-3 found in plant sources such as nuts and seeds, was associated with decreased risk of wheeze in Japanese children at 16- to 24-month-old (67). Taken together, maternal intake of n-3 PUFAs may have beneficial effects on preventing atopic disorders in offspring, including wheeze and asthma (63), although its preventive effect against food allergy is not clear (68). Whether these results reflect a generally healthier diet remains to be determined. The role of n-6 in offspring allergy will be discussed in section Dietary Supplements in Human Studies for maternal allergic influences.

#### Breastfeeding

##### Food allergen consumption

The recent clinical report by American Association of Pediatrics (AAP) proposes that no conclusions can be drawn regarding the effects of breastfeeding on preventing or delaying the development of food allergies in offspring (69), however, the report also recognizes that there is now evidence that early peanut introduction may prevent peanut allergy, as discussed below. The recent groundbreaking Learning Early About Peanut Allergy (LEAP) trial in the UK showed that introducing peanuts to infants at high risk for allergy at 4 to 10 months of age reduces the incidence of peanut allergy (34). The study recruited 834 infants with mild eczema with no egg allergy and infants with severe eczema, egg allergy, or both and subdivided based on peanut wheal responses by skin prick test into 0-, 1- to 4-, and >4-mm groups (35). All infants with mild eczema with no egg allergy showed no peanut-specific IgE sensitization, but 91% of the group with >4-mm peanut wheal responses exhibited peanut-specific IgE sensitization. The infants at the high risk for peanut allergy were randomly assigned to ingest or avoid peanut until 60 months of age (*N* = 640). Among the infants who were initially negative for skin-prick test (*N* = 530), the prevalence of peanut allergy at 60 months of age was considerably lower in the consumption group (1.9%) than those in the avoidance group (13.7%). Among the infants who were initially positive for skin-prick test (*N* = 98), the prevalence of peanut allergy was 10.6% and 35.3% in the consumption group and in the avoidance group, respectively (*p* = 0.004). Interestingly, levels of peanut-specific IgG4 were increased in the consumption group whereas levels of peanut-specific IgE were increased in the avoidance group. The study concluded that early introduction of peanut reduced the development of peanut allergy in high-risk children and that early peanut introduction does not prevent other allergies (asthma, seasonal rhinoconjunctivitis, perennial rhinoconjunctivitis, and eczema), sensitization to other food allergens (egg, milk) and aeroallergens, or reported allergic reactions to tree nuts and sesame (70). A follow-up Enquiring About Tolerance (EAT) trial in the UK with 1,303 infants demonstrated that early introduction of 6 allergenic foods [cow's milk, peanut, hard-boiled hen's egg, sesame, whitefish (cod), and wheat] was achievable by 6 months of age and did not affect breastfeeding (36). The study recruited exclusively breast-fed infants from 3 months of age and randomly assigned to standard introduction group (SIG) or early introduction group (EIG). The EIG was introduced 6 allergenic foods alongside continuous breastfeeding until at least 6 months of age. The study showed multiple allergenic foods can be safely introduced into infant diet without adverse influence on breastfeeding. The results failed to show better efficacy of early introduction in the prevention of food allergy to one or more of the 6 intervention foods: food allergy developed in 7.1 and 5.6% of the participants in the SIG and in the EIG, respectively (*p* = 0.32). However, in families that closely adhered to the challenging protocol, the prevalence of any food allergy was considerably lower in the EIG than in the SIG (2.4 vs. 7.3%, *p* = 0.01), as was the incidence of peanut allergy (0 vs. 2.5%, *p* = 0.003) and egg allergy (1.4 vs. 5.5%, *p* = 0.009). There was no significant effect with respect to the prevalence of allergy to milk, sesame, fish, or wheat. These studies suggest that the early introduction of all 6 allergic foods, although not easily achieved, may have beneficial effects in preventing food-allergen sensitization in high-risk children.

In addition to early direct food introduction to children, maternal consumption of potentially allergic foods during breastfeeding has been suggested to prevent food sensitization in offspring. A Canadian cohort study with 342 infants analyzed the association of maternal peanut consumption while breastfeeding, timing of direct peanut introduction to infants, and a combination of both, with peanut sensitization at 7 years of age (26). Breastfeeding and peanut consumption by mothers and infants were captured by repeated questionnaires during infancy. The lowest prevalence of sensitization with peanut was observed in infants whose mothers consumed peanut while breastfeeding and who were directly introduced peanut before 12 months of age (1.7%). Maternal peanut consumption with delayed introduction of peanut to infants beyond 12 months of age or maternal peanut avoidance with a direct introduction of peanut by 12 months of age was associated with an increased risk of peanut sensitization (*p* = 0.003) (15.1 and 17.6%, respectively). These results suggest that a combination, but not alone, of maternal peanut consumption while breastfeeding and direct introduction of peanut in the first year of life provides a significant protection from peanut sensitization. Although the readout was peanut sensitization and not challenge-proven peanut allergy, this study raises an important point that the combination of maternal and offspring factors together during breastfeeding period plays a critical role in the prevention of food allergy in offspring.

##### Allergen, allergen-specific Igs, and cytokine

Maternal food allergen consumption results in an appearance of allergen in breast milk, though not always. Ara h 2 and Ara h 6 are detectable in breast milk (71). Ara h 6 appeared in breast milk from non-atopic peanut-tolerant mothers as early as 10 min after peanut ingestion and continued being detected over a 24-h period (72). IC consisting of allergen and allergen-specific IgG and IgA were present in breast milk. Such human milk containing peanut allergens and IC induced a partial oral tolerance in peanut-sensitized young mice (72), indicating a protective role of milk-born allergen in reducing disease susceptibility in offspring. Microarray technology revealed that allergen-specific IgG and IgE antibodies are transmitted from maternal blood into breast milk and that breast milk IgG pattern mirrored the profile of IgG reactivity in maternal blood (73), which suggest a possibility that the transmission of allergen-specific Igs from mothers to offspring via milk may affect the development of allergy in neonates. Our recent study demonstrated that allergen-specific IgG in milk may play a crucial role in protection of offspring from food allergy as IC. Our study showed that ovalbumin (OVA)-specific IgG4 was detectable in 10 subjects among 16 breast milk samples from non-atopic mothers. OVA-IgG4-IC were detectable in 8 of 10 mothers who had OVA-IgG4 (74). Importantly, supplementation of humanized mice expressing human FcRn constitutively with human breast milk containing OVA-IgG-IC resulted in protection of those mice against food allergic responses, suggesting a critical role of maternal IgG-IC in breast milk in the induction of tolerance against food allergy. These results, together with our mouse studies as discussed below (section Breastfeeding in Mouse Studies for maternal protective influences), imply that the concept of IgG-IC contributing to protection against food allergy may extend to humans (74).

Maternal cow's milk avoidance was associated with lower levels of milk allergen-specific IgA and IgG4 antibodies and the development of cow's milk allergy in infants (75). High concentration of milk allergen-specific IgA in breast milk significantly reduced transcytosis of milk allergen through gut lumen, suggesting that IgA may prevents excessive, uncontrolled food antigen uptake in the gut lumen and thus in the prevention of cow's milk allergy. The Prediction of Allergies in Taiwanese Children (PATCH) birth cohort study assessed levels of secretary IgA (sIgA) in colostrum and stool from infants within 5 days after birth and at 2 and 4 months of age (76). sIgA concentration in colostrum was significantly higher in allergic mothers than those in non-allergic mothers (*p* = 0.01). Fecal sIgA concentration in breastfed infants was significantly higher in those in formula-fed infants (*p* <0.05). The association of breastfeeding with the increased levels of fecal sIgA in infants may suggest potential protective effects in early life.

A systematic review suggests a positive association between concentration of TGF-β1 or TGF-β2 in breast milk and a reduction in IgE titer and atopic inflammation in infancy and early childhood (77). In contrast, a prospective study with 398 mothers demonstrated an association of higher eczema risk with higher concentration of TGF-β2 in breast milk (OR, 1.04) at 6 months of age (78). Interestingly, detectable levels of IL-13 in colostrum were associated with lower parental-reported food allergy (OR, 0.10) at 6 months of age (78). High levels of IL-1β, IL-17, and C-C motif chemokine ligand (CCL) 17 (TARC) and low levels of C-X-C motif chemokine ligand (CXCL) 1 and thymic stromal lymphopoietin (TSLP) in breast milk were associated with a low risk of eczema at 0–3 years of age (79). Although the mechanisms are unknown and further large clinical studies are needed to confirm these results, allergens, IgG, IgA, and regulatory cytokines such as TGF-β in breast milk likely orchestrate to develop and maintain appropriate neonatal immune responses and may prevent adverse immunological outcomes in infants.

##### Breastfeeding duration, feeding modes, and maternal allergy

Although the beneficial effects of breastfeeding on global health of offspring are recognized, the effects of breastfeeding on the development of allergic diseases in offspring is controversial and remains largely elusive. Regarding the potential beneficial effects of breastfeeding duration on preventing allergies in children has been well-summarized in a recent review by American Academy of Pediatrics (69), although the available human data are still not sufficient to draw firm conclusions regarding various aspects of allergy prevention. Briefly, there is evidence that exclusive breastfeeding for 3 to 4 months decreases the development of eczema before 2 years of age, but there are no short- or long-term benefits of exclusive breastfeeding beyond 3 to 4 months for prevention of atopic diseases (69). Any duration of breastfeeding mode than 3 to 4 months is protective toward wheezing in the first 2 years of life (69). Some evidence suggests that longer duration of any mode of breastfeeding, including partial and exclusive breastfeeding, protects toward asthma even after 5 years of age (69). The role of breastfeeding duration in food allergies is much less clear. A cohort study in Taiwan enrolled 186 children demonstrated that exclusive or partial breastfeeding for longer than 6 months reduced milk sensitization in children at 1 and 1.5 years of age (30) compared to formula-fed children (OR, 0.37). A retrospective cohort study including 649 children with challenge-proven food allergy (31) demonstrated that every additional month of breastfeeding decreased a risk for food allergy by ~4% per month (OR, 0.96; *p* = 0.02). Within breastfed children, there was no significant association between the incidence of food allergy and direct breastfeeding vs. bottle-feeding (*p* = 0.27). Collectively, these studies indicate the association of prolonged breastfeeding with reduced risk of allergic sensitization and food allergy. Regarding feeding modes, a prospective birth cohort study in Canada with 3,296 children examined the effects of infant feeding modes on childhood asthma (32). Among direct breastfeeding alone, breastfeeding with some expressed breast milk, breast milk plus formula, or formula alone, direct breastfeeding was more protective against childhood asthma at 3 years of age as compared to formula-feeding. Indirect breast milk by other modes of infant feeding conferred intermediate protection. The differences in the preventive effects of direct breastfeeding vs. expressed breast milk may reflect a possible alternation of milk factors such as immune cells, cytokines, and microbiota. Maternal allergy may influence the effects of breastfeeding on allergic sensitization in children. A Canadian study that included 2,773 infants compared the effects of breastfeeding on offspring wheezing in the presence or the absence of maternal asthma (33). In the presence of maternal asthma, development of wheezing in infants was reduced by 62% with exclusive breastfeeding [adjusted rate ratio (aRR), 0.38] and by 37% with partial breastfeeding (supplemented with solid foods; aRR, 0.38) as compared to infants with no breastfeeding. Partial breastfeeding supplemented with formula was not protective for wheezing in infants (aRR, 0.89). Importantly, such association was not significant in the absence of maternal asthma. The role of breastfeeding duration, feeding modes, and maternal allergies in the development of offspring food allergy needs intensive future studies, however, these results above may favor the concept that longer period of breastfeeding confers protection against allergic diseases in infants.

#### Perspective

Maternal diet is not only an important factor for growth of fetus but also likely shapes neonatal immune responses and determines the susceptibilities of offspring to infectious diseases and allergies (80). Nutrition, allergens, Igs, cytokines, and also immune cells are transferred from mothers to neonates via placenta and during breastfeeding and may influence offspring allergy (Table 2). Direct breastfeeding may be beneficial to efficiently transfer protective factors such as microbiota from mothers to offspring. Human studies show conflicting results and whether and how maternal factors transferred *in utero*, breastfeeding, or both, exert preventive effects toward allergies in children (Tables 1, 2). Future cohort study and meta-analysis with systematic data of allergen consumption (maternal and offspring), immune active milk components together with environmental and genetic factors are needed to answer those questions.

**Table 2:** Maternal protective and risk factors for offspring allergy in human studies.

**Factors**	**Association/outcome**	**Effects**	**References**
**PRENATAL MATERNAL HEALTH AND ENVIRONMENT**			
Maternal iron concentration	Maternal serum concentration of ferritin status during the first-trimester of pregnancy was inversely correlated with risk of childhood wheeze and impaired lung function.	Protective (childhood wheeze)	(81)
Dexamethasone	Prenatal exposure of preterm infants to dexamethasone increased their susceptibility to allergic diseases, particularly asthma and allergic rhinitis.	Allergic (asthma, allergic rhinitis)	(82)
H2 blocker	Maternal exposure to H2 blockers or proton pump inhibitors during pregnancy increased the risk of developing asthma.	Allergic (asthma)	(83, 84)
Preeclampsia, pre-pregnancy overweight, and obesity	Preeclampsia, maternal pre-pregnancy overweight, and maternal obesity were associated with an increased risk of wheezing in offspring.	Allergic (wheezing)	(85)
Phthalate	· High levels of maternal exposure to phthalate during pregnancy reduced the number of Treg cells in cord blood as well as in children at 2 years of age. · Prenatal high concentrations of phthalate metabolites in maternal urine were significantly associated with a higher risk for atopic dermatitis in children until 3 years of age.	· Allergic (atopic dermatitis) · Allergic (atopic dermatitis)	(86)
Maternal adverse life events	Prenatal maternal adverse life events were suggested to link an increased risk for the development of atopic disorders in offspring.	Allergic (asthma, eczema)	(87)
Vaginal delivery	Protection toward atopy at later life, potentially reflecting the capacities of TGF-β1 to skew the production of IgE toward non-inflammatory isotypes IgG4 and IgA	Protective (atopy)	(49, 50)
Cesarean section	· Associated with decreased pro-inflammatory cytokine responses to TLR1/2 stimulation, followed by a higher abundance of bacterial colonization in the airway during late infancy, and increasing the risk of infantile wheezing · Lower levels of TGF-β1 in newborns	· Allergic (wheezing) · Allergic (atopy)	(48) (49)
Vitamin D	· 25% reduced risk of asthma and recurrent wheeze at 0–3 years of age by prenatal vitamin D supplementation based on combined analysis of VDAAT and COPSAC2010 · Maternal prenatal vitamin D supplementation was inversely associated with the development of food allergy. · Prenatal high concentration of serum maternal 25(OH)D was associated with an increased incidence of allergic airway disease in offspring at 20 to 25 years of age.	· Protective (asthma, wheezing) · Protective (food allergy) · Allergic (allergic airway disease)	(59) (62) (60)
Antibiotics	Both prenatal and child's use of antibiotics were associated with an increased risk of cow's milk allergy.	Allergic (cow's milk allergy)	(88)
**MATERNAL PUFAS**			
Fish oil, docosahexaenoic acid, and eicosapentaenoic acid	· Prenatal intake of oily fish containing higher docosahexaenoic acid and eicosapentaenoic acid as well as fish oil supplements showed protective effects on wheeze and asthma in offspring. · Prenatal fish oil supplementation reduced the severity of atopic eczema and the risk of egg sensitization in one-year-old offspring.	· Protective (wheezing, asthma) · Protective (atopic eczema, egg sensitization)	(65) (66)
α-linoleic acid	High prenatal intake of α-linoleic acid was associated with a reduced risk of wheeze in Japanese children at 16 to 24 months of age.	Protective (wheezing)	(67)
n-6 PUFA	Higher prenatal consumption of n-6 than n-3 during pregnancy was associated with higher risk of rhinoconjunctivitis in offspring at 5 years of age.	Allergic (rhinoconjunctivitis)	(89)
**MATERNAL ALLERGY**			
Cytokine	Prenatal cytokine production (cord-blood concentrations of IL-4, IFN-γ, and tumor necrosis factor) was associated with the development of atopy and asthma at 6 years of age.	Allergic (atopy and asthma)	(90)
Allergen sensitization	· Maternal asthma status did not confer additional risks for asthma or complications to black women and their infants, while maternal asthma status increased risk for both asthma and many complications to white and Hispanic. · Maternal sensitization to allergens was associated with reduced IFN-γ production from stimulated maternal peripheral blood mononuclear cells and elevated production of IL-13 in cord blood mononuclear cells from offspring. · Children from mothers with mild uncontrolled asthma, moderate-to-severe controlled asthma, and moderate-to-severe uncontrolled asthma showed higher prevalence of early-onset persistent asthma than those of mothers with mild controlled asthma. · Maternal asthma was a greater risk for offspring asthma than paternal asthma. · Maternal, but not paternal, total IgE levels correlated with elevated infant IgE levels and infant eczema. · Maternal asthma status did not affect the microbial populations in children.	· No association in black women (asthma) · Allergic in white and Hispanic women (asthma) · Potentially allergic (Th2 responses) · Allergic (asthma) · Allergic (asthma) · Allergic (IgE levels, eczema) · No association (microbiota)	(91) (92) (93) (94) (95) (96)
**BREAST MILK FACTORS**			
IgA in breast milk	· High concentration of milk allergen-specific IgA in breast milk reduced transcytosis of milk allergen through gut lumen. · sIgA concentration in colostrum was higher in allergic mothers than those in nonallergic mothers. · Fecal sIgA concentration in breastfed infants was significantly higher in those in formula-fed infants regardless of maternal allergy status.	· Protective (cow's milk allergy) · Potentially protective (sIgA levels in colostrum) · Potentially protective (fecal sIgA levels in infant)	(75) (76)
Maternal IgG transfer *in utero* and in breast milk	· Children of allergen-specific immunotherapy-treated mothers during pregnancy exhibited high levels of IgG antibodies and fewer positive skin prick test as compared with children of untreated mothers. · High levels of cord blood IgG antibodies to cat and birch were associated with less atopic symptoms in the children of allergic mothers during the first 8 years of life.	· Protective (grass allergy) · Protective (atopy)	(41) (42)
Allergens and IC in breast milk	· Human milk containing peanut allergens and IC induced a partial oral tolerance in peanut-sensitized young mice. · Allergen-specific IgG and IgE antibodies were transmitted from maternal blood into breast milk and that breast milk IgG pattern mirrored the profile of IgG reactivity in maternal blood. · Human breast milk containing OVA-IgG-IC reduced food allergic responses in mice.	· Protective (food allergy in mice) · Offspring outcome unknown · Protective (food allergy in mice)	(72) (73) (74)
Cytokine in breast milk	· Concentration of TGF-β1 or TGF-β2 in breast milk was associated with a reduction in IgE titer and atopic inflammation in infancy and early childhood. · Levels of IL-13 in colostrum were associated with lower parental-reported food allergy at 6 months of age. · High levels of IL-1β, IL-17, and TARC and low levels of CXCL1 and TSLP in breast milk were associated with a low risk of eczema at 0 to 3 years of age. · A prospective study with 398 mothers demonstrated an association of higher eczema risk with higher concentration of TGF-β2 in breast milk at 6 months of age. · Breast milk of allergic mothers contains higher levels of IL-4, RANTES, and IL-8 as compared to those of non-allergic mothers. · Levels of IL-1β, IL-10, and β-lactoglobulin-specific IgA were lower in breast milk from mothers of infants with cow's milk allergy than in breast milk from mothers of infants without cow's milk allergy.	· Protective (IgA levels, atopy) · Protective (food allergy) · Protective (eczema) · Allergic (eczema) · Potentially Allergic (offspring outcome unknown) · Allergic (cow's milk allergy)	(77) (78) (79) (78) (97) (98)

*25(OH)D, 25-hydroxyvitamin D; COPSAC, Copenhagen Prospective Studies on Asthma in Childhood; IC, immunecomplex; IFN, interferon; Ig, immunoglobulin; IL, interleukin; OVA, ovalbumin; PUFAs, polyunsaturated fatty acids; RANTES, regulated on activation, normal T cell expressed and secreted; sIgA, secretary IgA; TARK, C-C motif chemokine ligand (CCL) 17; TGF-β, tumor growth factor β; TLR, toll-like receptor; Treg, regulatory T cell; TSLP, thymic stromal lymphopoietin; VDAART, vitamin D antenatal asthma reduction trial*.

### Mouse Studies

Substantial numbers of mouse studies have reported preventive effects of maternal factors on development of allergic inflammation in offspring such as allergic airway inflammation and food allergic responses (Table 3). Offspring of dams sensitized with allergen or offspring nursed by allergen-sensitized or -exposed dams during breastfeeding show protection from the development of antigen-specific IgE and allergic inflammation following sensitization with allergen (74, 119). Many of these studies focus on the role of maternally transferred allergens and IgG via placenta and breast milk to offspring.

**Table 3:** Maternal protective and risk factors for offspring allergy in mouse models.

**Responsible factors**	**Strains**	**Treatment to dam**	**Dose/timing**	**Effects**	**References**
***IN UTERO***					
Allergen	BALB/c, DO11.10	· Oral administration · Oral administration	· High-dose OVA (9 mg) during the first week of pregnancy · Low-dose OVA (1.5 mg) during pregnancy · Low-dose OVA (1.5 mg) during pregnancy	· Suppression of OVA-specific IgE levels in 3-day-old, but not in 25-day-old, BALB/c offspring · Sustained inhibition of OVA-specific IgE levels both in 3- and 25-day-old BALB/c offspring ·*In utero* priming of offspring DO11.10-derived T cells	(99)
· B cells · Allergen-IgG-IC	C57BL/6, Mature B-cell-deficient μMT	Intranasal exposure	500 μg OVA before conception	· Induction of tolerance in C57BL/6 offspring against allergic airway inflammation · Abolished preventive effects against allergic airway inflammation in μMT · OVA-IgG1-IC bound to FcγR and promoted differentiation of Foxp3^+^ Treg.	(100)
Maternal IFN-γ	CD1	*i.p*. injection with IFN-γ	100 IU of recombinant IFN-γ during pregnancy	Maternal IFN-γ induced stronger protective effects when prenatally transferred *in utero* than when postnatally transferred via breast milk against allergic airway inflammation in offspring.	(101)
Maternal IgG and offspring IFN-γ	BALB/c, C57BL/6J, FcRn-deficient B6.129X1-*Fcgr^*ttm*1*Dcr*^*	Oral administration	100 mg OVA before conception	·*In utero* IgG transfer decreased the disease severity in BALB/c offspring. · Offspring of FcRn-deficient dam nursed by OVA-fed C57BL/6 WT dams showed protective effects against OVA-induced asthma-like symptom. · Anti-IFN-γ treatment of offspring during sensitization and challenge with allergen abolished the protection.	(102)
Maternal TLR signaling and offspring IFN-γ	BALB/c, C57BL/6 *Tlr2/3/4/7/9^−/−^*	Intranasal applications of freeze-dried *A. lwoffii* F78 suspended in PBS	10^8^ CFU of *A. lwoffii* F78 during prenatal period	· IFN-γ-dependent prevention of experimental asthma in BALB/c offspring · A significant reduction in acetylation levels of histone 4 of the *Il4* promoter after OVA challenge · The preventive effect was completely abolished in heterozygous offspring of *A. lwoffii* F78-treated *Tlr2/3/4/7/9^−/−^* dam.	(103, 104)
Maternal Th1-biased immunity	C57BL/6J	OVA with incomplete Freunt's adjuvant (*s.c*.) or alum (*i.p*.)	Preconceptionally immunized with 25 μg OVA with incomplete Freunt's adjuvant or 2 mg alum	· Offspring of dams with Th1-biased immunity showed reduced levels of OVA-specific IgE and airway eosinophilia compared with offspring of dams with Th2-biased immunity or naïve dams. · Suppression of allergic airway inflammation in offspring from Th1-biased dams was antigen-specific.	(105)
Short-chain fatty acid, especially acetate	C57BL/6	Fed with high-fiber diet or drinking water with acetate or propionate	· High-fiber diet: SF11-025 · 200 mM acetate or propionate	· Offspring of dams fed high-fiber diet or acetate failed to develop allergic airway disease. · Dams showed significant changes in microbiota composition, which was accompanied by an increase in high-acetate-producing Bacteroidetes and short-chain fatty acids in their feces and serum.	(106)
IL-4 signaling in asthmatic dams	BALB/c	Immunized with OVA/alum and exposed to OVA aerosol	Preconceptionally immunized with 5 μg OVA/1 mg alum and exposed to 3% (w/v) OVA aerosol during pregnancy	· Offspring of OVA-dams, but not of unsensitized dams, exhibited allergic airway inflammation in response to suboptimal sensitization with OVA/alum and OVA aerosol. · This model of maternal transmission of asthma susceptibility to offspring was not allergen-specific.	(107)
Maternal allergen-specific T cells	BALB/c	Adoptive transfer of DO11.10-derived T cells to naive dams	5 ×10^6^ DO11.10 T cells/mouse *i.p*. at 3 days before mating	· Higher responsiveness of offspring to suboptimal challenge of OVA · Donor T cells were identified in spleen and placenta of the recipient female dams but were not detectable in fetal tissues.	(108)
CD25 and GITR	BALB/c		· Depletion of CD25^high^ T cell population in 4-day-old newborn mice before induction of airway inflammation · Anti-GITR antibody treatment before induction of airway inflammation with suboptimal sensitization protocol · Anti-GITR antibody treatment after induction of airway inflammation	· Increased susceptibility to allergic airway inflammation in normal offspring · Substantial reduction in asthma symptoms in offspring of allergic dams · Significant attenuation of the susceptibilities to allergic airway inflammation in offspring of both naïve and asthmatic dams · Enhanced asthma-like allergic inflammation in normal offspring · Slight deterioration of allergic airway inflammation in the offspring of asthmatic dams	(109)
Skin barrier impairment	Offspring of male *Flg^*ft*/*ft*^/Tmem79^*ma*/*ma*^* and female C57BL/6	*i.p*. sensitization with OVA/alum and exposure to OVA aerosols (C57BL/6 WT dam)	5 μg OVA/1 mg alum and exposed to 3% (w/v) OVA aerosol before mating with unsensitized flaky tail male mice	· Increase in food allergen sensitization, serum mMCP1, and anaphylaxis to food allergen sensitization · Associated with higher levels of transcripts for CCL11, TSLP and IL-33 in skin, and TSLP in jejunum	(110)
**BREASTFEEDING**					
Allergen in breast milk	BALB/c	Airborne allergen exposure	0.5% OVA every other day during lactation	· Intact OVA and its degraded products were detectable in breast milk. · Offspring of OVA-exposed dams showed a significant protection against allergic airway inflammation, production of antigen-specific IgE, IgG1, and IgA antibodies, allergen-specific secretion of Th2 cytokines and IL-10 compared to offspring of unexposed dams. · The decrease in the disease susceptibility in offspring was independent on maternal Igs or IL-10.	(111)
Maternal TGF-β during breastfeeding and infant IFN-γ	BALB/c	Oral administration	2 mg OVA three times a week in the first, second, and third week of or throughout breastfeeding	· Oral tolerance induction in offspring were completely abolished in those nursed by dams treated with anti-TGF-β monoclonal antibodies. · Oral tolerance induction in offspring of OVA-fed dams was insufficient in 1-week-old neonates, accompanied by a reduction in gut barrier, retinaldehyde dehydrogenase expression by mesenteric lymph node CD103^+^ neonatal DCs. · One-week-old neonates were refractory to oral tolerance elicited by maternal allergen transfer via milk due to a physiological vitamin A deficiency. · Vitamin A supplementation rescued these neonatal defects and induced efficient tolerance in 1-week-old mice. · Oral tolerance was also dependent on offspring IFN-γ.	(111, 112)
IgG-IC	BALB/c	· OVA/alum *i.p*. · Exposure of OVA aerosols	· 10 μg OVA/2 mg alum, preconceptionally · 0.3% OVA during breastfeeding	· Induced a long-lasting allergen-specific protection from asthma. · OVA-IgG-IC transferred into offspring by FcRn was associated with the differentiation of Foxp3^+^ Treg cells in offspring. · OVA-specific DO11.10-derived T cells co-cultured with bone marrow-derived DCs proliferated and upregulated Foxp3 expression *in vitro* in the presence of breast milk from OVA-exposed dams or · OVA-IgG-IC purified from breast milk. · OVA-IgG-IC induced the proliferation of DO11.10-derived T cells at least 100-fold more efficiently than OVA alone. · Milk IC-mediated tolerance was more profound than the tolerance induced by milk-borne free allergen and did not require TGF-β, IgA, or FcγRIIb.	(18)
IgG1 and FcRn in offspring	B-cell deficient *Igh6^*tm*1*Cgn*^*, C57BL6/J, FcRn-deficient *Fcgrt*^−/−^	·*i.p*. injection with OVA/alum · OVA aerosol	· 25 or 8 μg OVA/2 mg alum before mating · 1% OVA dairy before mating and during embryonic days 11–17 of pregnancy	· Offspring nursed by B-cell deficient *Igh6^*tm*1*Cgn*^* mice failed to show protection against OVA-specific IgE production and OVA-induced allergic airway inflammation. ·*Fcgrt*^−/−^ offspring nursed by OVA-sensitized WT dams exhibited 1/1,000 to 1/10,000 lower levels of maternal OVA-specific IgG1 than those in similarly nursed *Fcgrt*^+/−^ or WT offspring and failed to show protection against OVA-specific IgE production and allergic airway inflammation.	(113, 114)
Amount of allergen directly taken by infant	C3H/HeJ	Peanut exposure by gavage	10 mg/mouse ground peanut three times a week for 4 weeks prior conception or preconceptionally and during pregnancy and breastfeeding	· No influence of maternal peanuts exposure during pregnancy on offspring peanut sensitization · Enhanced peanut-allergen uptake by CD11c^+^ DCs in Peyer's patch subepithelial dome in coadministration of peanut extract with breast milk from allergic dams as compared to coadministration of that from naïve dams · Oral administration of small dose (1 mg) of peanut extract to offspring with breast milk from sensitized or naïve dams during post-weaning period failed to enhance the induction of tolerance compared to those orally administered peanut extract alone in offspring.	(115)
Low dose allergen exposure during pregnancy and lactation	C3H/HeJ	Oral peanut/CT exposure by gavage	· 10 mg/mouse ground peanut/20 μg/mouse CT weekly and peanut exposure (50 mg/mouse) preconceptionally · 10 mg/mouse ground peanut/20 μg/mouse CT preconceptionally and during pregnancy and breastfeeding	· Offspring of dams preconceptionally sensitized with peanut/CT exhibited maternal IgG1-mediated anaphylaxis in response to oral peanut challenge. · Offspring were protected from anaphylaxis following first peanut challenge as well as active peanut/CT sensitization and oral peanut challenge.	(17)
Allergens and allergen- specific immunoglobulins	BALB/c	·*i.p*. injection of OVA/alum · Oral administration of OVA	· 50 μg OVA/1.3 mg alum twice at 2-week interval periconceptionally · 1% OVA in drinking water during breastfeeding for 2 weeks	· Offspring of OVA-sensitized and challenged dams showed the most significant reduction in food allergic responses, as indicated by a decrease in levels of diarrhea occurrence, OVA-specific IgE, *Il4* mRNA expression, and numbers of mucosal mast cells in proximal colon compared to those in offspring from naïve dams. · Offspring from dams unsensitized and challenged with OVA exhibited a partial but a significant reduction in food allergic responses compared to those from naïve dams. · Offspring from OVA-sensitized dams without oral OVA challenge showed comparable food allergic responses to offspring of naïve dams.	(116)
Oral TGF-β supplementation after weaning	BALB/c	·*i.p*. injection of OVA/alum · Oral administration of OVA	· 10 μg OVA/ 2 mg alum. twice at 1-week interval at 2 days before mating · 2 mg OVA by gavage three times a week during lactation	· Offspring of naïve dams nursed by OVA-sensitized dams showed a significant reduction in levels of diarrhea score, serum mMCP1, and OVA-specific IgE antibodies compared to naïve offspring nursed by naïve dams. · Breastfeeding by OVA-dams decreased the frequencies of allergic diarrhea in response to multiple oral OVA challenges in 6-week-old, but not in 13-week-old offspring. · Supplementation with TGF-β after weaning till 12-week prolonged protection against diarrhea and improved gut barrier in 13-week-old mice breastfed by OVA-dams.	(117)
Maternal OVA IgG-IC and offspring FcRn	BALB/c, FcRn-deficient *Fcgrt*^−/−^	· Epicutaneous sensitization ·*i.p*. injection of OVA-IgG1-IC	· 100 μg OVA preconceptionally and during pregnancy and breastfeeding · 100 μg OVA-IgG1-IC once weekly for 3 weeks during breastfeeding	· Maternal allergen sensitization through skin prevented food anaphylaxis, allergen-specific IgE production, serum IL-4, serum mMCP1, and intestinal mast cell expansion in BALB/c offspring. · The protective effects of food allergy by OVA-sensitized dam were abolished in *Fcgrt^−/−^*. · Offspring of naïve dams supplemented with OVA-IgG1-IC showed increase of serum OVA-IgG1-IC levels, OVA-specific Treg in mesenteric lymph node at weaning, and protection against food allergy.	(74)
Th2-biased epigenetic alteration	C3H/HeJ	Intragastric	10 mg peanut/20 μg CT weekly for 5 weeks and 200 mg peanut at 6 weeks before conception	Offspring of dams sensitized and challenged with peanut exhibited increased food allergic responses following suboptimal dose of oral peanut/CT sensitization and peanut challenge, associated with hypomethylation at CpG sites of *Il4* gene promoter.	(118)

*A. lwoffii, Acinetobacter lwoffii; CCL, C-C motif chemokine ligand; CFU, colony forming unit; CT, cholera toxin; DC, dendritic cell; FcγR, Fc gamma receptor; FcRn, neonatal Fc receptor; Foxp3, forkhead box p3; GITR, glucocorticoid-induced tumor necrosis factor receptor-family related receptor; IC, immunecomplex; IFN, interferon; Ig, immunoglobulin; IL, interleukin; i.p., intraperitoneal; IU, international unit; mMCP1, mouse mast cell protease I; OVA, ovalbumin; PBS, phosphate buffered saline; s.c., subcutaneous; TGF-β, tumor growth factor β; Th, helper T cell; TLR, toll-like receptor; Treg, regulatory T cell; TSLP, thymic stromal lymphopoietin; WT, wild type*.

#### Preconceptional and *in utero* Factors

##### Allergen, allergen-specific Igs, and cytokine

Several studies reported preventive effects on allergic sensitization and allergic inflammation in offspring born from dams sensitized with or exposed to allergen. Oral administration of wild-type (WT) dams with OVA during pregnancy has been shown to decrease allergen sensitization in offspring (99). Oral administration of dams with high-dose OVA (9 mg) during the first week of pregnancy resulted in a suppression of OVA-specific IgE levels in 3-day-old, but not in 25-day-old, offspring following *intraperitoneal* (*i.p*.) sensitization with OVA/alum and *i.p*. OVA injection (99). Oral administration of dams with low-dose OVA (1.5 mg) during pregnancy resulted in a sustained inhibition of OVA-specific IgE levels both in 3- and 25-day-old offspring. Fifty-seven percent of CD4^+^ T cells from offspring of OVA-fed DO11.10 dams (transgenic mice bearing OVA-specific T-cell receptor) proceeded into S phase 72-h after *in vitro* stimulation with OVA. In contrast, the majority of CD4^+^ T cells from offspring of unexposed DO11.10 dams were arrested in G_0_/G_1_ phase, suggesting that offspring T-cells are primed *in utero* by oral administration of dams with allergen. It is noteworthy that OVA was undetectable in amniotic fluid, placental serum, or breast milk from OVA-fed dams during pregnancy. In contrast, when dams were orally administered during the first week of breastfeeding, OVA was detectable in milk from OVA-fed dams in proportion to the dam's intake dose. Levels of maternal allergen-specific Igs were not examined in this study. These results suggest that oral exposure of dams to low-dose allergen during pregnancy may result in T-cell priming to allergen *in utero*, although whether maternal Igs are involved in this setting is not clear. Intranasal exposure of WT dams with OVA before conception resulted in the induction of tolerance in offspring toward allergic airway inflammation as well as allergen-specific Th2 responses following *i.p*. sensitization with OVA and alum and intranasal OVA challenge (100). Interestingly, the preventive effects against allergic inflammation were completely abolished in B-cell deficient μMT dams. The study proposes that OVA-IgG1-IC bind to CD16 and CD32 Fcγ receptors (FcγR) on dendritic cells (DCs) and promote an efficient allergen-priming by DCs to promote differentiation of forkhead box p3 (Foxp3)^+^ regulatory T cells (Treg) *in utero*, suggesting a role of allergen-IgG-IC in the induction of neonatal tolerance during pregnancy. More recent study demonstrated that anti-IgE antibody treatment of pregnant dams prevents allergen-specific IgE production in offspring following allergen sensitization (120).

Effects of maternal IFN-γ on development of offspring asthma were investigated in dams treated with IFN-γ (101). Dams were *i.p*. injected with IFN-γ during pregnancy, then offspring were fostered after birth and nursed by normal dams untreated with IFN-γ (IFN/Nor). Conversely, offspring of naïve dams were fostered and nursed by dams treated with IFN-γ (Nor/IFN) or untreated normal dams (Nor/Nor). Following OVA inhalation, both IFN/Nor and Nor/IFN offspring showed reduced numbers of eosinophils in bronchoalveolar lavage fluid, but only IFN/Nor offspring showed almost normal lung histology. IFN/Nor offspring also showed amelioration of passive cutaneous anaphylaxis. These results suggest that maternal IFN-γ induces stronger protective effects when prenatally transferred *in utero* than when postnatally transferred via breast milk against allergic airway inflammation in offspring.

The importance of IFN-γ in the protection of offspring against allergic inflammation was proposed in another study that demonstrated that transfer of maternal IgG to the offspring induced offspring tolerance against asthma-like phenotype (102). Offspring of BALB/c WT dams orally administered OVA before conception were protected from development of allergic airway inflammation induced by *i.p*. sensitization and intranasal challenge with OVA. Offspring of OVA-fed dams nursed by naïve dams were also protected, suggesting the role of *in utero* IgG transfer in decreasing the disease severity in offspring. Offspring of FcRn-deficient dams, unable to transport IgG through placenta, nursed by OVA-fed WT dams showed protective effects against OVA-induced asthma-like symptoms, indicating the contribution of IgG transfer also via breast milk to protection of offspring. Anti-IFN-γ treatment of offspring during sensitization and challenge with allergen abolished the protection. This study suggests that this maternal IgG-mediated suppression of allergic airway inflammation is dependent on offspring IFN-γ, indicating the synergistic effects of maternal-offspring factors in shaping neonatal responses to allergic diseases. How OVA administration to dams through usually tolerizing oral route results in generation of allergen-specific IgG was not clear in this study.

Exposure of dams with farm-derived, gram-negative bacterium *Acinetobacter lwoffii* F78 during prenatal period resulted in prevention of experimental asthma in their offspring, which was dependent on maternal TLR signaling (103). The follow-up study demonstrated that the preventive effects were dependent on offspring IFN-γ (104) and that offspring from dams exposed to *A. lwoffii* F78 showed a significant reduction in acetylation level of histone 4, a marker for an open chromatin structure, of the *Il4* promoter after OVA challenge. Since bacteria were not detectable in offspring, these data imply that prenatal exposure of dams to certain microbiota exerts protective effects in offspring against allergic responses through epigenetic regulation of cytokines.

One of the earlier studies compared the effects of maternal factors from dams with Th1 immune responses vs. dams with Th2 immune responses on allergic airway inflammation in offspring. Dams were preconceptionally immunized with OVA and complete Freund's adjuvant *subcutaneously* followed by a booster of OVA plus incomplete Freunt's adjuvant *i.p*. to induce Th1-biased immunity, or immunized with OVA and alum *i.p*. to induce Th2-biased immunity. Immunized dams were exposed to OVA aerosol before pregnancy then received recall OVA aerosol during pregnancy. Offspring of dams with Th1-biased immunity showed reduced levels of OVA-specific IgE and airway eosinophilia in response to immunization with OVA and alum (OVA/alum) and challenge with OVA aerosol compared with offspring of dams with Th2-biased immunity or naïve dams. Offspring of dams with Th1-biased immunity to bovine serum albumin did not show protection against development of OVA-specific IgE or OVA-induced airway inflammation, suggesting that the suppression of allergic airway inflammation in offspring from Th1-biased dams is antigen-specific. Although offspring of Th2-biased dams failed to show protection against OVA-induced airway inflammation, these offspring showed a trend toward lower levels of OVA-specific IgE as compared to offspring of naive dams (105). As Th1-biased dams were challenged with allergen before and during pregnancy but not during breastfeeding, these data suggest that maternal transfer of allergen and allergen-specific Igs *in utero* in the context of Th1 immune responses results in the resistance of offspring to allergic airway inflammation. It is possible that allergen-specific Igs continued to be transferred from Th1-biased dams to offspring through breast milk, and that these antibodies also contribute to prevent allergic inflammation in offspring, as shown in their follow-up study (113) as discussed in section Breastfeeding in Mouse Studies for maternal protective influences.

##### Microbiota

Animal models have evidently shown that the intestinal dysbiosis confers susceptibility to food allergy (121) and that certain bacteria such as *Clostridia* species that are protective from food allergy (122–124). Germ-free mice colonized with feces from healthy infants, but not from cow's milk allergic infants, protected against anaphylactic responses to a cow's milk allergen and that *Anaerostipes caccae*, a non-spore-forming, butyrate producing *Clostridial* species was critical for the protection against food allergy (123). Bacteriotherapy with *Clostridiales* species, impacted by dysbiosis in human infants with food allergy, suppressed food allergy in mice via MyD88/ROR-γt^+^ Treg cell pathway (124), indicating that microbiota critically regulate food allergy. A study reported that progeny of dams fed with high-fiber diet during pregnancy showed suppression of allergic airway inflammation (106). Dams fed with high-fiber diet showed significant changes in microbiota composition, which is accompanied by an increase in high-acetate-producing *Bacteroidetes* and short-chain fatty acids in their feces and serum. The increase in short-chain fatty acid, especially acetate, in dams during pregnancy induced Treg in offspring mediated by enhancing acetylation of Foxp3 promoter through inhibition of histone deacetylase 9 *in utero*. These reports suggest that maternal microbiota influences the susceptibility of offspring to allergies through epigenetic modification *in utero*.

Collectively, these observations indicate that maternal environment and maternal factors directly or indirectly affect offspring immune system during pregnancy likely contribute to the induction of tolerance. Potential preventive effects of *in utero* factors on offspring food allergy have been much less studied as compared to asthma models. Our study shows that offspring born from allergen-sensitized dams nursed by naïve dams exhibited a trend toward protection against food allergy (74), suggesting that *in utero* factors such as IC contribute to the reduced disease susceptibility, however, breastfeeding is essential to induce optimal tolerance induction. Investigations of the timing (prenatal, postnatal, or throughout), factors (allergen, Igs, IC, cytokine milieu, and microbiota), and dose of those factors in the interplay of pathogenic vs. regulatory responses in allergic diseases in offspring will be needed to understand this complex relationship.

#### Breastfeeding

In mouse studies, breastfeeding has been shown to be critical in the induction of tolerance toward allergic sensitization in postnatal period (74, 119). Similar to *in utero* period, maternal factors such as allergen, Igs, IC, and TGF-β in breast milk induce allergen-specific tolerance against allergic sensitization and inflammation. Maternal immune cells are present in breast milk and are transferred into offspring tissues (125). These factors may act individually or in synergy to prevent development of allergy in offspring through multiple mechanisms.

##### Allergen, allergen-specific Igs, and cytokine

Tolerance induction by allergen in milk has been the subject of many studies that use experimental asthma models. Aerosol OVA exposure of WT dams during lactation resulted in an allergen transfer to offspring via milk and the induction of allergen-specific tolerance toward allergic airway inflammation (111). Intact OVA and its degraded products were detected in breast milk from OVA-exposed dams during breastfeeding with the concentration in the similar range as those in human breast milk after consumption of egg or cow's milk (180 ± 20 ng/ml) (111). Offspring of OVA-exposed dams showed a significant protection against allergic airway inflammation, production of antigen-specific IgE, IgG1, and IgA antibodies, allergen-specific secretion of Th2 cytokines and IL-10 compared to offspring of unexposed dams. The decrease in the disease susceptibility in offspring was independent on maternal Igs or IL-10. Given that dams were *not sensitized* with allergen, i.e., in the absence of maternal allergen-specific Igs, it is not surprising that maternal Igs were not required for the induction of tolerance in this system. In contrast, the suppressive effects in offspring were completely abolished in those nursed by dams treated with anti-TGF-β monoclonal antibodies, indicating that maternal TGF-β during breastfeeding are required for the induction of tolerance. In mice, 1-week-old neonates were refractory to oral tolerance induced by maternal allergen transfer through milk due to a physiological vitamin A deficiency (112). Unsensitized dams were given OVA in the first, second, or third week of or throughout lactation and tolerance induction in offspring to OVA-induced allergic airway inflammation was examined. Oral tolerance induction in offspring of OVA-fed dams was insufficient in 1-week-old neonates, accompanied by a reduction in gut barrier, the expression of retinaldehyde dehydrogenase by mesenteric lymph node CD103^+^ neonatal DCs leading to inefficient T-cell priming, and serum retinol levels as compared to those in adult mice. Supplementation with vitamin A rescued these defects in neonates and induced efficient tolerance in 1-week-old mice. Oral tolerance was also dependent on offspring IFN-γ. These data may suggest maternal allergen transfer through milk together with vitamin A supplementation as possible interventions for prevention of allergy in the neonates (112). The same group next demonstrated that breastfeeding by preconceptionally sensitized WT dams with OVA/alum *i.p*. exposed to OVA aerosols during breastfeeding induced a long-lasting allergen-specific protection toward asthma. Dams sensitized and exposed to OVA exhibited OVA-IgG-IC in breast milk (18). Eighty-five percent of OVA in breast milk formed OVA-IgG-IC (18). OVA-IgG-IC transferred into offspring by FcRn was associated with the differentiation of Foxp3^+^ Treg cells in offspring. OVA-specific DO11.10-derived T cells co-cultured with bone marrow-derived DCs proliferated and upregulated Foxp3 expression *in vitro* with breast milk from OVA-exposed dams or OVA-IgG-IC purified from breast milk. OVA-IgG-IC promoted the proliferation of DO11.10-derived T cells at least 100-fold more efficiently than OVA alone. Milk IC-mediated tolerance was more profound than the tolerance induced by milk-borne free allergen and did not require TGF-β, IgA, or FcγRIIb (18). These studies suggest that milk-borne allergen protects offspring against asthma, and allergen as IC induces more efficient tolerance in neonates independently of TGF-β. Consistently, earlier studies indicated that when dams are sensitized with allergen, breast milk-induced tolerance in offspring require maternal B cell and offspring FcRn (105, 113, 114). Offspring nursed by B-cell deficient *Igh6*^*tm*1*cgn*^ mice failed to show protection against OVA-specific IgE production and OVA-induced allergic airway inflammation (113). The same group demonstrated that FcRn^−/−^ offspring nursed by OVA-sensitized WT dams exhibited 1/1,000 to 1/10,000 lower levels of maternal OVA-specific IgG1 than those in similarly nursed FcRn^+/−^ or WT offspring (114) and failed to show protection against OVA-specific IgE production and allergic airway inflammation. Collectively, these studies imply that allergen, IgG, and IC in breast milk as well as offspring FcRn are crucial for the induction of tolerance in allergic airway inflammation in offspring.

Regarding food allergy, maternal effects on protection of offspring toward food allergy were investigated in several mouse models. C3H/HeJ dams (lack a functional TLR4 that recognizes lipopolysaccharide) preconceptionally exposed to peanut exhibited variable levels of maternal Igs in sera and breast milk, though, maternal preconceptional peanut exposure or also during pregnancy and breastfeeding showed no influence on offspring peanut allergy induced by oral immunization with peanut and cholera toxin (CT) and oral plus *i.p*. peanut challenge (115). The efficacy of a direct oral tolerance induction in offspring was similar in offspring of dams exposed to peanut and in offspring of dams that did not consume peanut. Oral administration of crude peanut extract together with breast milk of peanut exposed dams onto 5-week-old offspring induced an enhanced peanut-allergen uptake by CD11c^+^ DCs in Peyer's patch subepithelial dome as compared to coadministration of peanut extract with breast milk from naïve dams. However, oral administration of small dose (1 mg) of peanut extract to offspring with breast milk from sensitized or naïve dams during post-weaning period failed to enhance the induction of tolerance compared to those orally administered peanut extract alone in offspring. The authors suggest that dose of dietary allergens contained in breast milk may not be adequate to induce oral tolerance in their system. These results support the recent recommendations of no restrictions in diet for pregnant and breastfeeding mothers. Human milk containing peanut allergens induced partial protection in young mice following peanut sensitization (72), supporting a protective role of milk-born allergens in reducing offspring disease susceptibility. A more robust immunization of C3H/HeJ dams with oral peanut/CT and peanut exposure during pregnancy and lactation resulted in reduction of peanut allergy in offspring (17). Offspring of dams preconceptionally sensitized with peanut/CT exhibited maternal IgG1-mediated anaphylaxis following oral peanut challenge (first exposure). The anaphylaxis reaction was also dependent on platelet activation factor, but not IgE, as maternal IgE was undetectable in offspring. Remarkably, when sensitized dams received peanut/CT also during pregnancy and lactation, offspring showed protection against anaphylaxis following first peanut challenge as well as direct peanut/CT sensitization and oral peanut challenge. These findings suggest that maternal immune responses during pregnancy and lactation regulate the reduction of disease susceptibility in offspring.

Another study compared the susceptibility to food allergic responses of offspring from OVA/alum-sensitized vs. unsensitized dams with and without maternal oral OVA challenge during breastfeeding (116). Offspring of OVA-sensitized and challenged dams showed the most significant reduction in food allergic responses, as indicated by decreased levels of diarrhea occurrence, OVA-specific IgE, *Il4* mRNA expression, and numbers of mucosal mast cells in proximal colon compared to those in offspring from unsensitized and unchallenged (naïve) dams. Offspring from dams unsensitized and challenged with OVA exhibited a partial but a significant reduction in food allergic responses compared to those from naïve dams. Offspring from OVA-sensitized dams without oral OVA challenge showed comparable food allergic responses to offspring of naïve dams, indicating that there is no increase in food allergy susceptibility of offspring by *in utero* factors from allergen-sensitized dams in this system. OVA-specific IgG was detectable in breast milk and serum of offspring from OVA-sensitized dams regardless of oral OVA challenge. OVA-specific IgA was detectable in breast milk only from OVA-sensitized and challenged dams. These results suggest that allergen-specific IgG and oral allergen exposure during breastfeeding resulting in production of allergen-specific IgA were required to induce an effective protection toward food allergy in offspring. These results suggest that even allergen-sensitized dams could have preventive effects on offspring allergies through breastfeeding. A recent study also demonstrated that allergen-sensitized dams protect offspring against food allergy through breast milk (117). Offspring of naive dams were fostered after birth by OVA-sensitized dams to avoid *in utero* effects then dams were orally challenged with OVA during breastfeeding. Offspring of naïve dams nursed by OVA-sensitized dams showed a significant reduction in levels of diarrhea score, serum mouse mast cell protease 1 (mMCP1), and OVA-specific IgE antibodies compared to naïve offspring nursed by naïve dams. The duration of oral tolerance elicited by breast milk toward food allergy was assessed in offspring of unsensitized dams nursed by dams preconceptionally immunized *i.p*. with OVA/alum then fed OVA during lactation. Breastfeeding by OVA-dams decreased the frequencies of allergic diarrhea in response to multiple oral OVA challenges in 6-week-old, but not in 13-week-old, offspring. TGF-β supplementation after weaning till 12-week prolonged protection toward diarrhea and enhanced gut barrier in 13-week-old mice nursed by OVA-dams. Lactation by allergen-sensitized dams together with offspring TGF-β supplementation may maintain long-lasting prevention of allergic diarrhea (117).

Previous studies reported the involvement of allergen-specific IgG or IgG-IC from allergen-sensitized mothers in the protection of offspring against allergic airway inflammation via breastfeeding and FcRn (18, 102, 114, 126). In line with these reports, our recent study critically elucidated the fundamental mechanisms by which IC in breast milk regulates the induction of tolerance in offspring toward food allergy (74). Dams were epicutaneously sensitized with OVA or a clinically relevant allergen peanut followed by epicutaneous sensitization and oral challenge of their offspring with the same allergen. Maternal allergen sensitization through skin prevented food anaphylaxis, allergen-specific IgE production, serum IL-4, serum mMCP1, and intestinal mast cell expansion in offspring. The induction of tolerance was mediated by neonatal FcRn-dependent transfer of maternal OVA IgG-IC via milk and induction of allergen-specific Treg cells in offspring.

Neonatal tolerance was induced by breastfeeding by OVA-sensitized dams or maternal supplementation with IgG-IC, suggesting an essential role of breast milk IgG-IC in the induction of tolerance. In addition to FcRn-dependent IgG-IC transfer, our work demonstrated that FcRn-dependent presentation of antigen by CD11c^+^ DCs was required for the induction of oral tolerance in offspring. Human breast milk from non-atopic mothers contained OVA-IgG-IC and was sufficient to induce tolerance in humanized FcRn mice, providing an important evidence for the potential relevance of our findings in human food allergy. Collectively, these findings demonstrate that interactions of offspring FcRn and maternal IgG-IC are critical in the induction of Treg cell responses that control food-specific tolerance in neonates, indicating the key role played by maternal allergen-specific Igs in milk in establishing tolerance that protects offspring from food allergy. These outcomes extend well beyond the previously defined roles of maternal antibodies and FcRn in stipulating passive immunity. Additionally, such food-specific IgG antibodies are induced during oral immunotherapy in humans and have been shown to act via FcγRIIb to suppress IgE-mediated hypersensitivity (127). Strategies that modulate maternal IgG responses might be beneficial for the prevention of food allergy in offspring.

#### Perspective

*In utero*, the timing and dose of allergen exposure, allergen-specific Igs, and ICs along with the combination and balance of cytokine milieu and microbiota profile that reflective of maternal diet likely regulate the potential preventive effects in offspring toward allergies. Increasing evidences represent the importance of breastfeeding in the induction of neonatal tolerance, which is also modulated by maternal allergens, allergen-specific Igs, ICs, as well as cytokine profile (Table 3). Allergen alone in breast milk may induce offspring tolerance in synergy with TGF-β. Breastfeeding by allergen-specific IgG-positive dams protect offspring against allergic sensitization and allergic responses, particularly when dams are exposed to allergen during breastfeeding. The induction of tolerance in offspring by IgG or IgG-IC is more efficient than allergen alone and does not require TGF-β, although supplementation of TGF-β may prolong the efficacy of IgG-IC-induced protection in offspring. In humans, increased allergy risk during early childhood indicates inadequate immune regulation in this life period. Both in prenatal and postnatal periods, it seems that maternal factors should interact with offspring immune system within specific time of window to successfully induce neonatal tolerance. More mechanistic studies are needed to elucidate the precise molecular mechanisms by which maternal factors modulate allergen-specific Treg cells and tolerance in offspring. For example, a particular DC subset and Fc receptors that are responsible for the induction of Treg cell responses remain elusive. An inhibitory Fc receptor FcγRIIb and allergen-specific IgG have been shown to promote food tolerance (127, 128), but whether this receptor also plays a role in the context of maternal tolerance transfer needs to be examined. These issues will be the important subjects of future studies.

## Maternal Influences Over Offspring Allergy

### Human Studies

The early appearance of childhood allergies and the rise in their prevalence suggest that genetics and changes in early-life exposures *in utero* may increase the predisposition. In this section, we focus on the potential maternal risk factors influence on development of offspring allergy, including genetic variance, diet, immunomodulating factors, as well as health status and environmental exposure.

#### *In utero* Factors

##### Genetics

A family history of allergy has been shown to be a risk factor for development of allergies in offspring (129). Common genetic factors driving allergic diseases including atopic dermatitis, asthma, and allergic rhinitis have been suggested (130, 131). Regarding food allergy, the first large-scale genome-wide association study (GWAS) identified 10 loci associating with allergic sensitization as indicated by the presence of allergen-specific IgE against common environmental allergens and self-reported allergic symptoms (132). The report suggests that allergic heritability is associated more with the severity of allergic symptoms and a general tendency for allergic sensitization (sensitization against any environmental allergen) than with sensitization against one specific allergen (132, 133). A recent GWAS in 2017 has identified five susceptibility loci for the disease; the clade B serpin (SERPINB) gene cluster, the cytokine gene cluster, the filaggrin gene, the C11orf30/LRRC32 locus, and the human leukocyte antigen region were identified using a multi-ethnic database (134). All of the identified loci are involved in epithelial barrier function or immunological regulation, highlighting the role of both mechanisms in the development of food allergy.

Twin studies have provided the strong evidences for the genetic determination of allergic diseases. A systematic review of twin and sibling studies suggests that the overlaps in loci between different allergic diseases may partly explain allergic co-morbidities and the concept of the “atopic march” (135). A Swedish cohort study including 25,306 twin aged 9 or 12 years showed that asthma and allergic diseases of childhood are greatly heritable. In this study, the heritability of any childhood asthma was 0.82. For the other allergic diseases including wheezing, hay fever, atopic eczema, and food allergy, the range was ~0.60–0.80 (136). A twin study of eosinophilic esophagitis indicated the frequencies in both monozygotic and dizygotic twins were significantly higher than in siblings (137), suggesting that eosinophilic esophagitis also shows the heritability. These heritability estimates are variable in different studies, probably due to differences in study designs and target populations.

Racial disparities are also identified in allergic diseases. It has been shown that African Americans are sensitized to food and aeroallergens more frequently than whites (138). The analysis of genetic markers for the ancestry suggests that the racial disparities and ancestry are associated with elevated levels of food allergen-specific IgE (139). The effects of maternal asthma on racial/ethnic disparities in obstetrical and neonatal complications were tested (91). Analysis of data acquired from white (*N* = 110,603), black (*N* = 50,284), and Hispanic (*N* = 38,831) singleton for joint effects of maternal race/ethnicity and asthma on the odds of obstetrical/neonatal complications indicated that maternal asthma status did not confer additional risks for asthma or complications (preeclampsia, neonatal intensive care unit admissions, maternal hemorrhage, small for gestational age, and apnea) to black women and their infants, while maternal asthma status increased risk for both asthma and many complications to white and Hispanic (91). A meta-analysis study demonstrated that maternal asthma is a greater risk for offspring asthma than paternal asthma (OR, 3.04 vs. 2.44, *p* = 0.037) (94). Maternal, but not paternal, total IgE levels correlated with elevated infant IgE levels and infant eczema (95). The effect of parental allergy on food allergy in children is yet to be discovered.

While these studies have been useful and will continue to identify novel genes for food allergy and other allergies, future approaches to investigate the mechanisms by which such genetic factors play a role in maternal influences over food allergy in offspring will improve our understanding of the disease mechanisms and produce potential opportunities for prevention and treatment of the disease.

##### Food allergen consumption

In contrast to potential preventive effects of allergen exposure that we discussed earlier in this article, an increased risk by maternal consumption of allergenic foods has been previously proposed. A study enrolled 503 infants 3 to 15 months of age with likely milk or egg allergy without previous diagnosis of peanut allergy and investigated the association of maternal peanut intake with peanut sensitization in offspring. They identified a dose-dependent association of maternal peanut ingestion during the third trimester of pregnancy with peanut sensitization in offspring (12). There was no significant effect of maternal peanut consumption during the first or second trimester of pregnancy or during breastfeeding on offspring peanut sensitization in this study. Among infants never breastfed, frequent ingestion of peanut during pregnancy was strongly associated with peanut sensitization in offspring. These results suggest that maternal consumption of peanut during pregnancy is a potential risk factor of offspring food sensitization, although oral food challenges were not performed due to the age of the group. Earlier study that investigated 13,971 children of non-atopic and atopic mothers reported no association of maternal peanut consumption during pregnancy with challenge-proven peanut allergy in preschool children (13). Effects of maternal and infant food-allergen avoidance on development of early onset atopy were investigated in a prenatally randomized, controlled trial of infants of atopic parents (28). Children of prophylactic-treated group (*N* = 103) (maternal avoidance of egg, cow's milk, and peanut during the third trimester of pregnancy and breastfeeding period, infant use of casein hydrolysate for supplementation or weaning, and avoidance of solid food for 6 months, avoidance of cow's milk, corn, citrus, and wheat for 12 months, and avoidance of egg, peanut, and fish for 24 months) showed a lower prevalence of atopy at 12 months compared to those in control group (*N* = 185) with unrestricted diets, occurring from reduced food-associated atopic dermatitis, urticaria, and/or gastrointestinal disease by 12 months (*p* = 0.007) and any positive food skin test by 24 months (*p* = 0.019) in the prophylactic-treated group. However, the suppression of prevalence of food allergy and milk sensitization was significant only before 2 years of age and none of the prevalence in food allergy, atopic dermatitis, allergic rhinitis, asthma, any atopic disease, and serum IgE levels were differed at 7 years of age (29), indicating that maternal and offspring avoidance of allergenic foods is not effective in prevention of allergic diseases in offspring.

##### Allergen-specific Igs and cytokine

As to Ig transmission, the mechanisms responsible for the acquisition of maternal antibodies other than IgG are not fully understood. Cord blood IgE has been proposed as a predictive marker for allergic diseases (140). However, it is suggested that cord blood allergen-specific IgE does not reflect intrauterine sensitization but rather is the result of maternal IgE transfer to the fetus, which is mediated by FcRn that accelerates the transepithelial transport of IgE in the form of IgE-IgG-IC (141, 142). The significance of this IC in allergy development in offspring has not been investigated. A birth cohort study has shown the association of prenatal cytokine production (cord-blood concentrations of IL-4, IFN-γ, and tumor necrosis factor) with the development of atopy and asthma at 6 years of age. The study suggests that neonatal cytokine production patterns are significant predictors of a following risk for allergy and asthma (90). Another study indicated that maternal allergen sensitization is associated with reduced IFN-γ production from stimulated maternal peripheral blood mononuclear cells and increased production of IL-13 in cord blood mononuclear cells from offspring (92).

##### Microbiota

Increasing evidences highlight the role of microbiota in development of allergies [reviewed in (143)]. Children born by caesarian section are suggested to be more susceptible to develop allergies, as appropriate exposure to microbiome of newborns through natural birth may be required for the maturation of their immune system in early life. Consistently, use of antibiotics by both mothers and children were associated with an increased risk of cow's milk allergy (88). In 2018, it is reported that maternal asthma status did not alter the microbial populations in the children. They showed that maternal asthma was, however, a key modifier between the microbiome and asthma risk, which pointed to susceptibility to host-microbial interactions particularly for these children (96). The study suggests that such susceptibility could occur from an inborn immune deviation determined by maternal asthma status (144).

##### Dietary supplements

Early-life exposures such as infection, environmental pollutants and nutrients provided via the mother may act upon the developing fetal immune system and lead to allergic diseases. Studies investigating the association of maternal nutrients such as vitamin D, PUFAs, and folate (or the synthetic form, folic acid) have been equivocal (145). Eicosanoids, signaling molecules made from PUFAs play a role in inducing inflammations. High levels of eicosanoids are associated with an increase in the prevalence and severity of allergic diseases (63). The association of high levels of maternal dietary intake of linoleic acid, a common n-6 fatty acid, both during pregnancy and after birth with an increased risk of allergic diseases in children has been demonstrated. A Finnish study showed that higher consumption of n-6 than n-3 during pregnancy was associated with higher risk of rhinoconjunctivitis in offspring at 5 years of age (89). A study on German schoolchildren in Leipzig in 1995-1996 (*N* = 2,334) showed that schoolchildren who consumed linoleic acid-rich margarine exhibited a higher risk of hay fever as compared to schoolchildren who did not consume margarine (146). Similarly, a longitudinal study on Australian schoolchildren in 1980 showed higher levels of margarine consumption and lower levels of butter consumption among children with atopic disease as compared to non-atopic children (147). Since n-6 and n-3 compete for metabolism by the same enzyme, too high intake of n-6 would reduce the amount of enzyme available for n-3, decrease the n-6/n-3 ratio, and result in the production of more proinflammatory modulators that may lead to inflammation including asthma (148). Maternal serum concentration of Fe states (ferritin concentration) during first-trimester of pregnancy was inversely correlated with risk of childhood wheeze and impaired lung function (81). Beneficial effects of maternal consumption of specific nutrients on preventing offspring are still largely unknown.

##### Maternal health and environment

Maternal health condition including control of maternal allergies and maternal exposure to certain chemicals are important factor to influence neonatal health condition. Maternal severe asthma without using inhalant corticosteroid treatment related to reduced birth weights of female children (149). However, prenatal exposure of preterm infants to dexamethasone, a type of corticosteroid medication, increased their susceptibility to allergic diseases, especially asthma and allergic rhinitis (82). In a Danish cohort study that employed 675,379 singletons, children from mothers with mild uncontrolled asthma, moderate-to-severe controlled asthma, and moderate-to-severe uncontrolled asthma showed higher prevalence of early-onset persistent asthma than those of mothers with mild controlled asthma (93), indicating that the severity and control of maternal asthma increase risk for offspring asthma. Although poorly controlled maternal asthma is associated with augmented risk of development of allergy, the use of corticosteroid during pregnancy is only recommended when there are no alternatives and benefit outweighs risk. Maternal exposure to H2 blockers or proton pump inhibitors also increase the risk of developing asthma, especially in children whose mothers purchased these medication more than 3 times within 2 months prior to conception and during pregnancy (83, 84). Preeclampsia, maternal pre-pregnancy overweight, and maternal obesity were associated with an increased risk of wheezing in offspring in a pooled analysis of 14 birth cohort studies (85). High levels of maternal exposure during pregnancy to phthalate reduced the number of Treg cells in cord blood as well as in children aged 2 years (86). Furthermore, high concentrations of phthalate metabolites in maternal urine were significantly associated with a higher risk for atopic dermatitis in children until 3 years of age (OR, 2.21; *p* = 0.026). The study suggests that the reduction in Treg cells in offspring by maternal exposure to phthalate may facilitate the development of atopic dermatitis in early childhood.

Maternal adverse life events such as separation or divorce, marital problems, problems with the children, pregnancy problems, involuntary job loss experience, involuntary job loss of partner, money problems, a residential move, death of a close relative, and death of a close friend during pregnancy are suggested to link an increased risk for atopic disorders in offspring (87).

In addition to genetic predispositions linked to allergies, these studies clearly suggest a critical involvement of maternal health and environmental factors in the development and manifestation of allergy in offspring. Optimal management of maternal health might be essential in determining the allergic predisposition and clinical symptoms in offspring.

#### Breastfeeding

##### Allergen, allergen-specific Igs, and cytokine

Maternal and environmental factors modify the milk composition and may facilitate development of allergic diseases in neonate. Dietary allergens such as peanut, egg, and cow's milk proteins have been detected in human milk (72, 150–152) and may induce symptoms in already sensitized infants (152). However, their role in early sensitization vs. tolerance induction in offspring is still unclear. Airborne allergens from house dust mite are contained in human breast milk that may facilitate sensitization to food allergens in mice (153). Infants with cow's milk allergy have specific IgE that cross-reacts with endogenous human protein in breast milk (154), potentially suggesting that cow's milk allergy may trigger allergic march because cow's milk proteins could be the first foreign proteins recognized by infant. Breastfeeding may increase a risk for atopic eczema and food allergy in breast-fed infant (155). As we discussed earlier, food avoidance by allergic mothers was associated with offspring allergy (75). Lower levels of milk-specific IgG4 and IgA in allergic mothers who have eliminated milk from their diet during breastfeeding were associated with the development of cow's milk allergy, implying the potential protective roles of maternal food allergen consumption against offspring allergies via food-specific IgG4 and IgA in breast milk. Concentrations of chemoattractant factors were higher in breast milk of allergic mothers than in those of non-allergic mothers. The breast milk of allergic mothers contained higher levels of IL-4, regulated on activation, normal T cell expressed and secreted (RANTES) and IL-8 as compared to those of non-allergic mothers (97). Another study reported that the levels of IL-1β (*p* = 0.001), IL-10 (*p* = 0.04), and β-lactoglobulin-specific IgA (*p* = 0.005) were lower in breast milk from mothers of infants with cow's milk allergy than in breast milk from mothers of infants without cow's milk allergy (98). The component of breast milk of allergic mothers may be different from that of non-allergic mothers, although the effects of these differences on offspring allergies are poorly understood.

#### Perspective

Evidences indicate that genetics are one of the most critical factors that determine the susceptibility of allergic sensitization and severity of allergic symptoms in the offspring. The impacts of genetics are likely amplified by maternal nutrition and environmental factors including drug usage and exposure to harmful chemicals during pregnancy. Breastfeeding by allergic mothers may be another modifier of allergic reactions in offspring. Allergen, Ig, and IC transferred *in utero* and via breast milk likely play important roles in promoting the susceptibility of offspring by regulating the balance of Th2 vs. Treg cell responses. It is still unclear which factor is crucial to determine susceptibility to allergic sensitization and clinical manifestation of allergic inflammation in offspring (Table 2). Further prospective birth cohort studies and meta-analysis are needed to elucidate crucial risk factors for allergic sensitization in offspring transferred from allergic mothers.

### Mouse Studies

In this section, we focus on literatures demonstrated the increased susceptibility of offspring from allergic dams to allergic inflammation and discuss the role of maternal transmission of allergies to offspring.

#### Preconceptional and *in utero* Factors

##### Cytokine, T cell, and genetic factors

Mouse models of experimental allergies have been utilized to dissect how maternal factors affect the susceptibility of offspring to allergic inflammation. The susceptibility of offspring from asthmatic dams to development of allergic airway inflammation has been intensely studied. Hamada et al. preconceptionally immunized BALB/c WT dams with OVA/alum and exposed to OVA aerosol during pregnancy. Offspring of OVA-dams, but not of unsensitized dams, exhibited allergic airway inflammation in response to suboptimal sensitization with OVA/alum and OVA aerosol, suggesting maternal transfer of asthma risk to offspring (107). This model of maternal transmission of asthma risk to offspring was not allergen-specific (107), as offspring of asthmatic dams sensitized and challenged with OVA also showed higher responsiveness to casein-induced allergic airway inflammation. Blocking of IL-4 signaling in asthmatic dams by administration of anti-IL-4 antibodies before mating abolished the increased susceptibility of offspring from asthmatic dams to suboptimal sensitization protocol, suggesting that the maternal transfer of asthma risk to offspring is IL-4 dependent (107). Th2 responses in asthmatic dams skewed fatal and neonatal immunity toward Th2 and were associated with the increased asthma susceptibility in offspring that persisted through the young adulthood (~3 or 6 weeks) in offspring (156, 157). Adoptive transfer of DO11.10-derived T cells to naive dams prior to mating resulted in higher responsiveness of offspring to suboptimal challenge of OVA (108). Donor T cells were identified in spleen and placenta of the recipient female dams but were not detectable in fetal tissues, suggesting that the factors produced by maternal allergen-specific T cells mediated the response. Naïve offspring of asthmatic dams showed significantly high levels of spleen cellularity due to an increase in CD4^+^CD25^+^Foxp3^−^ effector T cell population (109). Interestingly, depletion of CD25^high^ T cell population by anti-CD25 antibody (clone PC61) in 4-day-old newborn mice before induction of airway inflammation by suboptimal sensitization protocol resulted in the increased susceptibility to allergic airway inflammation in normal offspring. In contrast, depletion of CD25^high^ T cell resulted in a substantial reduction in asthma symptoms in offspring of allergic dams. Anti-glucocorticoid-induced tumor necrosis factor receptor-family related receptor (GITR) antibody (clone DTA-1) treatment before induction of airway inflammation with suboptimal sensitization protocol significantly attenuated the susceptibilities to allergic airway inflammation in offspring of both naïve and asthmatic dams. Contrary, anti-GITR antibody treatment after induction of airway inflammation enhanced the development of asthma-like allergic inflammation in normal offspring, but induced only a slight deterioration of allergic airway inflammation in the offspring of asthmatic dams. The authors suggest that the increased asthma susceptibility in offspring of asthmatic dams can be prevented by modulating maternal Th2 responses as well as neonatal regulatory responses with anti-CD25 (pre-sensitization in asthma-prone offspring) or -GITR antibodies (pre-sensitization in both normal and asthma-prone offspring). As OVA-dams were *not* exposed to OVA during lactation, these data may indicate that lack of allergen transfer through milk together with maternal sensitization is critical for maternal transmission of asthma risk to offspring (51, 118, 156, 158–160).

Skin barrier impairment also affects the susceptibility to food allergy in offspring born from allergen-sensitized dams. WT dams were *i.p*. sensitized with OVA/alum and exposed to OVA aerosols before mating with unsensitized flaky tail (ft) male mice that exhibit an impaired skin barrier due to homozygous mutations of filaggrin (*Flg*) and mattrin (*Tmem79*^*ma*^) genes (*Flg*^*ft*/*ft*^*/Tmem79*^*ma*/*ma*^). Resulting *Flg*^*ft*/+^*/Tmem79*^*ma*/+^ offspring showed an increase in food allergen sensitization, serum mMCP1, and anaphylaxis to food allergen sensitization through tape stripped skin with peanut extract and *Alternaria alternate* extract followed by food allergen challenge compared to those born from unsensitized WT dams (110). These food allergic responses were associated with higher levels of transcripts for CCL11, TSLP and IL-33 in the skin, and TSLP in the jejunum. This study suggests the possibility that responses to food allergens of neonatal mice are dependent on genetic defects in cutaneous barrier function and on exposure to environmental allergens.

#### Breastfeeding

Both maternal allergic sensitization and allergen consumption seem to alter the immunological components of breast milk that affect offspring allergy. Using the mouse model of maternal asthma transfer as discussed above, Leme et al. demonstrated the role of breast milk from allergen-sensitized dams in the development of allergy in offspring (161). Both offspring from asthmatic dams nursed by unsensitized dams and offspring from unsensitized dams nursed by asthmatic dams showed enhanced airway hyperresponsiveness and allergic airway inflammation after allergen sensitization compared to offspring from and nursed by unsensitized dams. The authors suggest an equal transmission of asthma to the offspring either *in utero* or through breast milk.

With regards to food allergy, Song et al. demonstrate that maternal peanut allergy increases susceptibility to offspring allergy in link with Th2-biased epigenetic alterations in a mouse model of peanut allergy (118). C3H/HeJ dams were sensitized with oral peanut/CT and challenged before mating. Offspring of dams sensitized and challenged with peanut exhibited increased food allergic responses following suboptimal dose of oral peanut/CT sensitization and peanut challenge, associated with hypomethylation at CpG sites of *Il4* gene promoter. Interestingly, unlike their previous study demonstrating that maternal exposure to peanut during pregnancy and lactation reduces peanut allergy risk in offspring (17), dams did not receive sensitization during pregnancy and breastfeeding in this study. These results suggest that maternal sensitization may increase food allergy susceptibility in offspring in the absence of maternal allergen exposure during pregnancy and breastfeeding. Together, these studies suggest that maternal immune responses during lactation are critical for the reduction of food allergy susceptibility in offspring, consistent with the increasing numbers of studies demonstrating that breastfeeding by allergen-sensitized dams results in the induction of tolerance rather than the maternal risk transfer (as discussed in section Breastfeeding in Mouse studies for maternal protective influences).

#### Perspective

In some mouse studies, offspring of asthmatic dams have been shown to be more susceptible to asthma than offspring of non-allergic dams. However, the efficacy of breastfeeding by asthmatic dams in ameliorating allergic symptoms have not been thoroughly investigated. It is currently unclear whether offspring of allergic dams who did not receive breast milk may exhibit higher susceptibilities to allergies and move severe allergies than breastfed offspring. Multitudes of factors likely contribute to the maternal life during pregnancy and breastfeeding period, including allergen exposure and allergic responses. Depending on doses, timing, and number of antigen challenges, multiple factors in breast milk could transfer mediators that either increase or decrease offspring responses to allergen (Table 3). In the recent dramatic shift in recommendations from avoidance of common food allergens to early ingestion to prevent the development of allergy in offspring support the concept that early allergen introduction, potentially through breastfeeding, may decrease the risk of offspring allergies. Investigations of exact timing and dose of allergen exposure as well as early critical factors are necessary for development of safe and effective strategies for high-risk infant.

## Conclusions

Evidences from human and mouse studies suggest that maternal allergen exposure and resulting maternal immune responses that are transferred through placenta and breast milk likely influence offspring immune responses toward those allergens. Such interactions are regulated by genetic factors and environmental factors (maternal microbiota, nutrients, and health) to determine the induction of tolerance vs. allergy outcome in offspring through Th1, Th2, and Treg cell responses.

Current human data are not sufficient to draw firm conclusions regarding the role of breastfeeding in preventing or delaying the development of food allergies in offspring (69), however, there is now evidence that early introduction of peanuts may prevent peanut allergy (Table 1). Mouse studies suggesting maternal transfer of asthma risk to offspring *lack* maternal allergen exposure during pregnancy and breastfeeding. Other studies suggest maternal transmission of tolerance to offspring against asthma through breast milk allergen and IC. Although the investigations using experimental food allergy within this area are still developing, those studies consistently suggest that maternal sensitization and exposure with allergen induce tolerance in offspring, especially via breast milk. These results from experimental asthma and food allergy models may seem inconsistent at first glance, yet they are consistent in that maternal allergen sensitization plus allergen exposure *during pregnancy and breastfeeding*, which are likely tolerogenic periods, are critical in the induction of tolerance vs. allergies in offspring (Table 3). Those studies, including our own, are in line with several recent findings in humans as (1) maternal exposure to food allergens reduces allergy in offspring in humans, (2) recent decisions to cancel recommendations of allergen avoidance during pregnancy and breastfeeding, and (3) early food introduction potentially decreasing the risk of food allergy development. The last finding is particularly relevant to the results from mouse studies, as food allergen transfer via milk may be the first food exposure for the infant.

Development of a feasible and optimal instruction for maternal and offspring consumption of potentially allergenic food and nutrient during pregnancy and breastfeeding are urgently needed. Thorough analysis of human amniotic fluid and breast milk component based on challenge-proven food allergies in offspring in combination with genetic analysis may help with development of such instruction. Consideration of dose, frequencies, and period of food consumption during pregnancy and breastfeeding together with nutrient or immunological supplementation of breast milk may help with the novel strategies for prevention and treatment of food allergy in offspring.

The precise mechanisms by which maternal factors, such as allergen exposure during breastfeeding, exert a lasting effect on determining the susceptibility of offspring toward allergy need further investigations. Findings in mouse studies need to be carefully interpreted due to the differences between mice and humans. However, the powers of animal models are being able to investigate specific questions that are impossible or unethical to address in human subjects. To date, epidemiological studies have been performed at population levels and not at personal levels. Combined future research of translational and clinical science will help us with understandings of the role of maternal factors on offspring food allergy and will lead to effective preventive care of the disease.

## Author Contributions

TF, SL, YN, and MO wrote the paper. TF and MO arranged and revised the manuscript. SK and MO conceived and supervised the paper. TF, SL, and MO have agreed to be accountable for all aspects of the manuscript and in securing that questions related to the accuracy of any part of the manuscript. MO has given a final approval of the version of the manuscript to be published.

### Conflict of Interest Statement

The authors declare that the research was conducted in the absence of any commercial or financial relationships that could be construed as a potential conflict of interest.

## Abbreviations

AIT: Allergen-specific immunotherapy
aRR: Adjusted rate ratio
CT: cholera toxin
DC: dendritic cell
FcRn: neonatal Fc receptor
Foxp3: forkhead box p3
IC: immunecomplex
IFN: interferon
Ig: immunoglobulin
IL: interleukin
i.p.: intraperitoneal
mMCP1: mouse mast cell protease I
OR: odds ratio
OVA: ovalbumin
PUFAs: polyunsaturated fatty acids
sIgA: secretary IgA
TGF-β: tumor growth factor β
Th: helper T cell
Treg: regulatory T cell
WT: wild type.
